# SMART AGING: ENGAGING OLDER ADULTS TO GUIDE SENSOR DEVELOPMENT TO SUPPORT AGING IN PLACE

**DOI:** 10.1093/geroni/igad104.1908

**Published:** 2023-12-21

**Authors:** Elinor Schoenfeld, Tracy Trimboli, Givenchy Ayisi-Boahene, Patricia Bruckenthal, Erez Zadok, Shelley Horwitz, Fan Ye

**Affiliations:** Stony Brook School of Medicine, Stony Brook, New York, United States; Stony Brook University, Stony Brook, New York, United States; Stony Brook University, Stony Brook, New York, United States; Stony Brook University, Stony Brook, New York, United States; Stony Brook University, Stony Brook, New York, United States; Stony Brook University, Stony Brook, New York, United States; Stony Brook University, Stony Brook, New York, United States

## Abstract

Over 80% of older adults want to live independently in their own homes and communities, maintaining quality of life, autonomy, and dignity as they age. We are using community engaged research methods to aid in developing in-home cost-conscious remote sensing technologies to support older adults age in place. To understand their needs, we engaged older adults in discussions on home-based sensing technologies. We used visuals and demonstrations to facilitate discussions, showing participants our sensor prototypes and a vignette describing the challenges an older adult and the family face managing a chronic condition. Participants voiced their interest in monitoring for select health conditions and situations when either they or the person(s) they care for are home alone. Discussants raised concerns about personal security/privacy, loss of independence, ethics of data collection and sharing, and being overwhelmed by collected data. Discussions have provided valuable feedback to help us develop a sensor system that is flexible enough to accommodate individuals in different life stages and comfort levels, with different home environments, levels of expendable income, and support structures. As a result, we have developed a system that uses nonvisual, non-wearable sensing that measures respiration and heart rates, and indoor location tracking to monitor the health and wellbeing of users. During this session, we will provide detailed results from our community discussions, and discuss the continuing role for community engagement as we move forward with sensor development and testing.

